# Evaluation of a Powered Ankle-Foot Prosthesis during Slope Ascent Gait

**DOI:** 10.1371/journal.pone.0166815

**Published:** 2016-12-15

**Authors:** Christopher A. Rábago, Jennifer Aldridge Whitehead, Jason M. Wilken

**Affiliations:** 1 Center for the Intrepid, Department of Rehabilitation Medicine, Brooke Army Medical Center, JBSA Fort Sam Houston, Texas, United States of America; 2 The Extremity Trauma and Amputation Center of Excellence, JBSA Fort Sam Houston, Texas, United States of America; Northwestern University, UNITED STATES

## Abstract

Passive prosthetic feet lack active plantarflexion and push-off power resulting in gait deviations and compensations by individuals with transtibial amputation (TTA) during slope ascent. We sought to determine the effect of active ankle plantarflexion and push-off power provided by a powered prosthetic ankle-foot (PWR) on lower extremity compensations in individuals with unilateral TTA as they walked up a slope. We hypothesized that increased ankle plantarflexion and push-off power would reduce compensations commonly observed with a passive, energy-storing-returning prosthetic ankle-foot (ESR). We compared the temporal spatial, kinematic, and kinetic measures of ten individuals with TTA (age: 30.2 ± 5.3 yrs) to matched abled-bodied (AB) individuals during 5° slope ascent. The TTA group walked with an ESR and separately with a PWR. The PWR produced significantly greater prosthetic ankle plantarflexion and push-off power generation compared to an ESR and more closely matched AB values. The PWR functioned similar to a passive ESR device when transitioning onto the prosthetic limb due to limited prosthetic dorsiflexion, which resulted in similar deviations and compensations. In contrast, when transitioning off the prosthetic limb, increased ankle plantarflexion and push-off power provided by the PWR contributed to decreased intact limb knee extensor power production, lessening demand on the intact limb knee.

## Introduction

Walking up slopes is significantly more demanding than walking on level surfaces, requiring greater joint power production [1] through increased joint ranges of motion (ROM) [2] for the purpose of raising and propelling the body’s center of mass up the slope [3]. During able-bodied (AB) gait up a slope, many adaptations are observed at the ankle [4]. For example, when transitioning off the limb (terminal stance through preswing), trailing limb ankle plantarflexor musculature contributes significantly to forward and upward propulsion [1, 2]. When transitioning onto the limb (initial contact through midstance), leading limb ankle dorsiflexion allows the knee and hip to rotate over the foot at midstance, continuing forward motion of the body [1, 2]. In midstance, hip extensors contribute to the support moment and also aid in pulling the body up the slope [1, 2].

In the absence of an intact ankle, individuals with unilateral transtibial amputation (TTA) must rely on prosthetic ankle-foot devices to meet the demands of walking up a slope. Unfortunately, the mechanical limitations of many prosthetic devices, such as reduced ROM and plantarflexion power compared to an intact ankle, can cause significant gait deviations during slope ascent [5–8]. Specifically, individuals with TTA ascending slopes demonstrate decreased walking velocities compared to AB individuals, increased demands on the intact ankle when transitioning onto the prosthetic limb, and reduced propulsion from the prosthetic limb when transitioning off the prosthesis [6–8].

Vickers and colleagues found that older adults with TTA who used a solid-ankle-cushioned-heel prosthetic ankle-foot (SACH) ascended a 5° slope at half the speed of age-matched controls and spent less time in prosthetic limb stance [7]. The SACH exhibited a limited ankle ROM of 10° with reduced power generation compared to AB individuals. To compensate for these limitations, the TTA group significantly shortened their stride lengths and relied on greater proximal and intact limb muscle activation to accomplish this task. The authors suggested these gait deviations and compensations could be reduced if prosthetic feet were capable of ROM and power generation similar to a healthy, intact ankle-foot complex [7].

In a study to investigate propulsion as individuals with TTA walked up a slope with an energy-storing-returning prosthetic ankle-foot (ESR), a net power absorption was observed over the gait cycle [5]. The ESR did provide energy return when transitioning off the prosthetic limb; however, push-off power and body center of mass propulsion was substantially less than that provided by the intact limb. This study further confirmed that a conventional ESR was not capable of producing the push-off power necessary to lessen demand on residual musculature.

In a more recent study, the gait of individuals with TTA was evaluated as they walked up a 7.5° slope using a microprocessor-controlled prosthetic ankle-foot (Össur, Proprio-Foot®) [6]. This prosthetic ankle-foot used accelerometer data to position itself in dorsiflexion prior to prosthetic limb initial contact. Compared to a neutral position, dorsiflexion allowed for greater rotation of the shank over the prosthetic ankle-foot resulting in increased prosthetic limb knee flexion. As individuals transitioned onto the prosthetic limb, increased prosthetic limb knee flexion was associated with a greater utilization of prosthetic limb knee extensors to lift the body’s center of mass upward and forward on the slope. Compared to a neutral position, prosthetic ankle-foot dorsiflexion also resulted in reduced ROM and power production from the trailing intact ankle-foot. Thus, positioning of the prosthetic ankle-foot in dorsiflexion appeared to lessen the demand on the intact limb when transitioning onto the prosthetic limb [6]. Despite the ability to position in dorsiflexion, this prosthetic ankle-foot functioned like an ESR when transition off the prosthetic limb with reduced plantarflexion and push-off power compared to an intact ankle. The reduction in propulsive power partly contributed to the reduction in walking velocity observed in the TTA group relative to AB individuals.

In contrast to an ESR, a microprocessor-controlled, powered prosthetic ankle-foot device (PWR; BiOM®, BiOM, Bedford, MA) has demonstrated push-off power generation peaks at or above intact ankle-foot values during incline [9], level ground [9–11], and stair [9, 12] ambulation. While walking up a 5° slope, individuals using a PWR had 53% greater step-to-step transition work relative to an ESR when transitioning off the trailing prosthetic limb [9]. The increased push-off provided by the PWR reduced mechanical energy losses of the leading intact limb on the slope. In contrast to level ground walking, push-off by the PWR on the 5° slope did not reduce metabolic rates relative to those observed with an ESR [9]. However, active push-off power and additional ankle ROM provided by the PWR was reported to contribute to an increase in preferred walking velocity on incline [9], level [9, 10], and uneven [13] surfaces. During level ground walking, a power absorption compensation at the prosthetic limb knee was observed when using the PWR [10]. Peak vertical force and loading rate in the intact limb decreases as walking speeds increases when using the PWR on level ground [11]. On uneven surfaces, a small, significant decrease in prosthetic limb knee flexion during early stance was observed with the PWR, but no additional lower limb deviations were exhibited in joints proximal to the ankles. During stair ascent, compensations made by bilateral hip extensors persisted even with increases in push-off power and peak plantarflexion provided by the PWR compared to an ESR [12].

While powered plantarflexion appears to be beneficial in many walking conditions, it is unclear if compensations will persist or new ones will arise when walking up a slope. It is unknown if the PWR would have a negative or beneficial effect when transitioning off or onto the prosthetic limb. The purpose of this study was to evaluate the effect of additional ankle plantarflexion and push-off power generation provided by a PWR on gait compensations exhibited by individuals with TTA during 5° slope ascent. When compared to AB individuals walking up a slope, we hypothesized that individuals with TTA performing the same task using an ESR would demonstrate decreased walking velocity, increased power generation in the intact ankle when transitioning onto the prosthetic limb, and decreased propulsion from the prosthetic limb when transitioning off the prosthesis [6–8]. Secondly, we hypothesized that active ankle plantarflexion and push-off power provided by a PWR while transitioning off the prosthetic limb would reduce compensations at the intact knee and hip. In addition, we also investigated gait compensations exhibited by the same individuals with TTA using their conventional ESR during 5° slope ascent. We hypothesized that when transitioning onto the prosthetic limb, reduced PWR dorsiflexion would result in ankle compensations at the intact limb similar to those observed with an ESR.

## Methods

### Participants

Thirteen individuals with unilateral TTA between the ages of 18 and 45 years old were recruited for this study. Ten participants were included for analysis as two participants were lost to follow-up and one participant had incomplete data. Prior to study enrollment, participants were screened to ensure they met the following inclusion criteria: walking without an assistive device for at least two months, ability to walk for a minimum of 15 consecutive minutes, pain less than 4/10 on a visual analogue scale, normal ROM in all intact joints, and free from concomitant injuries which would hinder walking ability. Their data has been previously reported for level-ground gait [10] and stair ambulation [12].

A matched AB group (n = 10) with no history of musculoskeletal or neurological impairment was established from preexisting data based on age, height, weight, and gender. Study approval was obtained from the Brooke Army Medical Center’s Institutional Review Board and informed written consent obtained from all participants.

### Prostheses Setup

Participants in the TTA group used an ESR as their primary prosthesis. To ensure that these participants would have a functioning prosthetic limb at all times, a separate limb was created for the PWR (BiOM®, BiOM, Bedford, MA) with a duplicate socket made from the ESR limb mold. All prosthetic alignment and fitting was performed by an experienced prosthetist. The alignments of both prosthetic devices were optimized for level ground walking and utilized patient feedback. Tuning of the PWR to include ankle joint power production, push-off timing, toe-off ankle angle, net non-conservative work, and foot stiffness was performed by a BiOM company representative to patient preference within a range of two standard deviations relative to data collected from AB individuals. Detailed descriptions of the BiOM and its control mechanisms can be found in the literature [14–16]. Participants wore their same footwear during each prosthetic device condition.

### Experimental protocol

**Fig 1:**
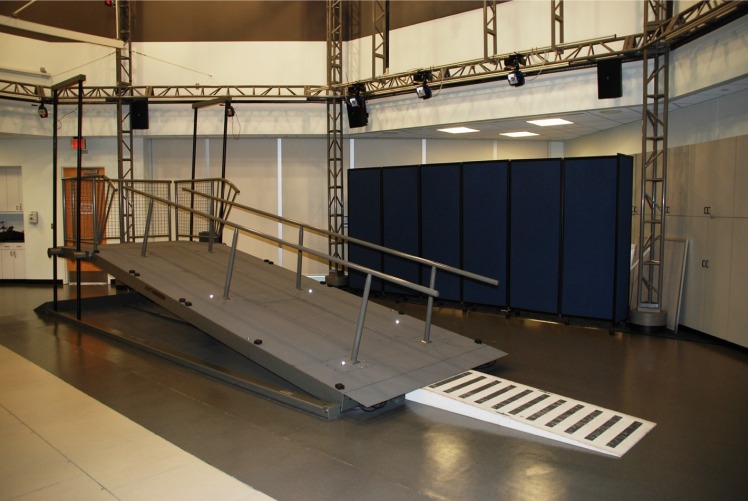
Picture of 5m long instrumented slope that participants walked up.

Temporal spatial step parameters and lower extremity joint kinematics and kinetics were measured during a biomechanical gait analysis to characterize slope ascent ambulation. The AB group attended a single gait analysis session. Their measures served as a representative of uninjured gait and used in the detection of gait abnormalities (i.e. deviations and compensations) in the TTA group. Participants with TTA attended two separate gait analysis sessions using their: 1)ESR and 2) PWR. Participants with TTA were given three weeks to acclimate to the PWR. During data collections, participants walked up a 5m long, 5° sloped ramp (Fig 1). They walked at a controlled velocity which was determined for each participant using leg length and a dimensionless Froude number of 0.16 [17]. Scaling of gait speed was performed to increase the ability to detect kinematic, kinetic, and temporal spatial group differences associated with function rather than leg length differences [18]. Participants were given a real-time auditory signal (Cortex, Motion Analysis Corp., Santa Rosa, CA) to control their velocity within 5% of the target velocity. A biomechanical gait analysis was also performed with participants walking at a self-selected velocity. Self-selected velocity data is presented in the S1 and S2 Tables and S1 Fig.

Motion capture collection procedures were similar to those previously reported [12]. A six degree-of-freedom marker set consisting of 57 reflective markers was used to collect kinematic data from 13 body segments [19]. Marker trajectories were recorded at 120 Hz with a 26 camera optoelectronic motion capture system (Motion Analysis Corp., Santa Rosa, CA). Ground reaction forces were collected at 1200 Hz using two force plates embedded into the surface of a custom built ramp (AMTI, Watertown, MA). A minimum of five strides with clean force plate strikes from each limb were obtained.

### Data Analysis

Marker trajectories and analog force plate data were filtered with a 4th order low-pass Butterworth filter (6 and 50 Hz cut-off frequencies respectively). Anatomical coordinate system definitions for all segments were determined in accordance with International Society of Biomechanics standards [20]. Visual 3D software (C-Motion Inc., Rockville, MD) was used to calculate joint angles using an Euler angle approach [Cardan rotation sequence of sagittal (z), frontal (x), transverse (y)] by comparing the orientation of distal and proximal joint segments. Joint moments and powers were calculated via inverse dynamics from marker and force plate data and resolved in the proximal segment‘s coordinate system [19]. Joint moments and powers were normalized to body mass. Kinematic and kinetic data were time-normalized to 100% gait cycle. Peak values for each kinematic and kinetic parameter of interest were extracted using a custom MATLAB program (Mathworks Inc., Natick, MA). Temporal spatial parameters were also calculated. The right limb of the AB group was used as the reference control for TTA group comparison.

Statistical analyses were performed using SPSS software (v. 19 IBM Sommers, NY). Mean and standard deviation calculations characterized group performance and variability of the dependent measures. Independent t-tests were used to identify between group differences in demographic and anthropometric data. Significance was set at p < 0.05.

Independent t-tests were used to determine temporal-spatial, kinematic, and kinetic data differences between the AB group and TTA groups’ intact and prosthetic limbs for each prosthetic device. A Bonferroni-Holm correction was performed for these four comparisons. The Bonferroni–Holm method uses a step-down approach to account for multiple comparisons by arranging p-values from the smallest to the largest and comparing them to sequential significance cutoffs [21]. A correction factor accounting for four comparisons was applied with the smallest p-value cutoff set to 0.05/4 = 0.0167.

Repeated measures 2x2 ANOVAs (limb x device), using subject as a random variable, were used to determine limb effects (intact, prosthetic) and device effects (ESR, PWR) on temporal-spatial, kinematic, and kinetic measures within the TTA group. Estimated marginal means post-hoc tests were used to delineate significant differences in these measures at p <0.05.

## Results

Participant demographics and anthropometrics are presented in Table 1. The TTA group was on average older than the AB group (p = 0.004) with no significant differences in height or weight between the groups. Limb lengths were on average 7cm longer (p = 0.037) for the TTA group compared to AB group (Table 1). Scaling walking velocities to limb length resulted in a controlled walking velocity mean approximately 11% greater (p = 0.001) for the TTA group compared to the AB group (Table 2). The TTA group walked at self-selected velocities approximately 12% and 18% faster than the AB group with the ESR (p = 0.002) and PWR (p = 0.004), respectively (Table 2). There was no statistical difference between the calculated controlled walking velocities and the velocities individuals self-selected. There were few differences between the controlled and self-selected velocity conditions. Temporal spatial, kinematic, and kinetic data at the controlled walking velocity are presented in the main text with self-selected velocity data presented in the S1 and S2 Tables and S1 Fig.

**Table 1:** Participant demographics and prescribed ESR devices.

TTA Group (n = 10)								AB Group (n = 10)					
ID	Sex	Amputated Side	Age (yrs)	Height (m)	Weight (kg)	Leg Length (cm)	Prescribed ESR	ID	Sex	Age (yrs)	Height (m)	Weight (kg)	Leg Length (cm)
1	M	R	32	1.93	102.0	108.0	Re-Flex VSP	1	M	21	1.83	103.9	95.5
2	M	L	38	1.80	99.0	101.0	Re-Flex VSP	2	M	23	1.87	101.8	100.0
3	M	R	29	1.93	108.9	113.0	FlexFoot	3	M	32	1.98	106.3	100.0
4	M	R	29	1.78	93.2	95.0	Re-Flex VSP	4	M	21	1.81	84.5	93.0
5	M	L	22	1.87	96.4	104.0	Renegade	5	M	21	1.82	93.4	95.0
6	M	R	38	1.70	97.7	92.5	Renegade	6	M	29	1.74	100.2	91.0
7	M	R	26	1.83	87.7	98.0	Re-Flex VSP	7	M	21	1.78	82.0	90.0
8	F	L	34	1.65	85.5	86.0	Renegade	8	F	19	1.67	84.3	89.0
9	M	R	29	1.83	93.2	92.5	Re-Flex VSP	9	M	24	1.73	93.2	87.0
10	M	R	25	1.93	97.5	107.0	Pathfinder	10	M	22	1.73	99.5	88.0
Mean ± SD			30.2 ± 5.3**ᵡ**	1.83 ± 0.10	96.1 ± 6.8	99.7 ± 8.4**ᵡ**				23.3 ± 4.1	1.80 ± 0.09	94.9 ± 8.8	92.9 ± 4.5

Abbreviations: Female (F), Identification number (ID), Left (L), Male (M), Right (R)

**ᵡ** Significantly different from AB values(p < 0.05)

**Table 2:** Temporal-spatial measures at a controlled velocity walking up a 5° slope. Measures are shown for the right limb of the able-bodied group and the intact and prosthetic limbs of the TTA group. The control and self-selected walking velocities are also shown.

Measures	Able-Bodied	TTA Intact Limb		TTA Prosthetic Limb	
		ESR	PWR	ESR	PWR
Stance Time (s)	0.74±0.06	**0.73±0.03ᶲ**	0.74±0.04	**0.70±0.03ᶲ**	0.73±0.03
Step Length (m)	0.73±0.05	**0.75±0.06ᶲ**	**0.75±0.07ᶲ**	**0.81±0.05ᵡᶲ**	**0.84±0.07ᵡᶲ**ᵑ
Step Time (s)	0.60±0.04	0.57±0.03	**0.58±0.03ᶲ**	0.58±0.02	**0.59±0.03ᶲ**
Stride Length (m)	1.47±0.11	1.56±0.10	1.59±0.13	1.56±0.11	1.60±0.14
Swing Time (s)	0.44±0.03	**0.42±0.02ᶲ**	0.43±0.02	**0.45±0.03ᶲ**	0.44±0.02
Controlled Velocity (m/s)	1.23±0.07	**1.36±0.07ᵡ**	**1.36±0.11ᵡ**	**1.36±0.07ᵡ**	**1.36±0.11ᵡ**
Self-Selected Velocity (m/s)	1.18±0.06	**1.32±0.10ᵡ**	**1.39±0.18ᵡ**	**1.32±0.10ᵡ**	**1.39±0.18ᵡ**

Note: Data are mean ± SD

**Bold:** Significant values

**ᵡ** Significantly different from the AB limb (p < 0.0125)

**ᶲ** Significantly different from the contralateral limb of the same prosthetic condition (p < 0.05)

**ᵑ** Significantly different from the same limb of the ESR condition (p < 0.05)

### Temporal Spatial

The primary temporal spatial deviation associated with prosthetic device use was significantly longer step lengths with the prosthetic limb (ESR and PWR) compared to the AB limb (p = 0.001; Table 2). Prosthetic limb step length increased by 4% (p = 0.330) with the PWR compared to the ESR. Significant inter-limb deviations were also seen in step length, stance time, and swing time with the ESR (p < 0.0125; Table 2). TTA group participants took 8% longer (p < 0.04) steps with their ESR limb compared to their intact limb. They also spent less time (p = 0.001) in stance and more time (p = 0.001) in swing with the ESR limb compared to the intact limb. During PWR use, TTA group participants took 12% longer (p < 0.001) steps with their PWR limb compared to their intact limb. Unlike with ESR use, there were no significant inter-limb deviations in stance and swing times when using the PWR. Step time with the PWR limb was slightly longer (p = 0.013) compared to the intact limb.

### Ankle Angle

All participants utilized a heel-toe strategy when contacting the ramp. Deviations in the TTA group were marked by significantly less prosthetic ankle ROM (ESR: 44% and PWR: 27%; p < 0.001) and greater intact ankle ROM (ESR: 20% and PWR: 22%; p < 0.001) compared to the AB group (Table 3A). Average ankle sagittal ROM in the prosthetic limb increased toward AB values by approximately 5° (p < 0.02) with the PWR compared to the ESR (Table 3A).

When transitioning onto the prosthetic limb, the PWR limb demonstrated significantly more (p < 0.007) peak plantarflexion at loading response compared to the AB and ESR limbs. During both device conditions, peak ankle plantarflexion at initial swing was significantly greater (p < 0.001) in the trailing intact limb compared to AB values.

**Table 3:** Peak joint angles, moments, and powers for the ankle, knee, and hip at a controlled velocity walking up a 5° slope. Measures are shown for the right limb of the able-bodied group and the intact and prosthetic limbs of the TTA group.

(A) Kinematic Parameters Peaks	Able-Bodied	TTA Intact Limb		TTA Prosthetic Limb	
		ESR	PWR	ESR	PWR
**Ankle angle (°)**	** **	** **	** **	** **	** **
Plantarflexion—Loading response	-1.08±3.46	**-4.50±4.42ᶲ**	**-0.65±4.09ᶲᵑ**	**-0.53±4.28ᶲ**	**4.61±2.95ᵡᶲᵑ**
Dorsiflexion—Terminal stance	16.20±3.06	16.56±2.89	**15.69±2.25ᶲ**	18.23±2.55	**13.88±1.97ᶲᵑ**
Plantarflexion—Initial swing	15.38±3.05	**21.18±4.41ᵡᶲ**	**22.69±4.88ᵡᶲ**	**-4.67±3.18ᵡᶲ**	**8.57±4.47ᵡᶲᵑ**
Sagittal range of motion	31.57±4.40	**37.74±5.09ᵡᶲ**	**38.43±4.82ᵡᶲ**	**17.73±4.42ᵡᶲ**	**22.87±3.96ᵡᶲᵑ**
**Knee angle (°)**					
Flexion—Initial contact	9.34±6.08	17.25±8.73	**5.78±6.62ᵑ**	14.26±9.32	**3.42±5.49ᵑ**
Flexion—Loading response	19.14±5.12	**29.81±7.34ᵡᶲ**	**18.78±4.68ᶲᵑ**	**18.78±9.61ᶲ**	**10.75±4.94ᵡᶲᵑ**
Extension—Terminal stance	-0.67±3.45	2.77±4.34	**-1.89±5.35****ᵑ**	4.37±8.84	**-3.21±5.33ᵑ**
Flexion—Swing	60.65±3.67	**60.19±6.69ᶲ**	**56.91±5.08ᶲ**	**64.40±2.61ᶲ**	**60.51±3.56ᶲᵑ**
Sagittal range of motion	62.56±3.21	57.97±6.34	**60.67±4.01ᶲ**	60.85±8.55	**64.81±5.38ᶲ**
**Hip angle (°)**					
Flexion—Loading response	39.31±4.47	**53.82±7.97ᵡ**	**44.96±4.54ᵡᶲᵑ**	**55.28±7.96ᵡ**	**50.08±5.40ᵡᶲᵑ**
Extension—Preswing	-8.85±5.70	**0.88±6.45ᵡ**	**-4.01±6.14ᶲᵑ**	**3.37±9.10ᵡ**	**0.28±7.21ᵡᶲ**
Flexion—Swing	37.91±3.59	**50.62±8.11ᵡ**	**42.86±5.93ᶲᵑ**	**54.25±6.12ᵡ**	**49.33±4.61ᵡᶲᵑ**
Sagittal range of motion	48.39±5.12	53.18±3.92	49.54±4.74	52.84±6.25	**50.76±6.14ᵑ**
**Pelvis angle (°)**					
Max Anterior tilt	11.70±4.68	**21.56±7.69ᵡ**	**19.33±3.91ᵡᶲ**	**21.93±6.94ᵡ**	**20.79±4.16ᵡᶲ**
Min Anterior tilt	7.92±4.94	**16.39±7.50ᵡ**	**14.68±3.96ᵡᶲ**	**16.34±7.36ᵡ**	**15.42±3.88ᵡᶲ**
Sagittal range of motion	3.79±1.06	5.16±1.97	4.65±1.69	5.60±2.22	5.37±1.97
**(B) Kinetic Parameter Peaks**					
				
**Ankle Moments (Nm/kg) & Powers (W/kg)**					
Dorsiflexion Mom—Loading response	-0.18±0.05	**-0.15±0.08ᶲ**	**-0.19±0.12ᶲ**	**-0.23±0.13ᶲ**	**-0.43±0.21ᵡᶲᵑ**
Plantarflexion Mom—Terminal stance	1.58±0.14	**1.69±0.38ᶲ**	**1.93±0.55ᶲ**	**1.49±0.34ᶲ**	**1.55±0.51ᶲ**
Power Abs—Loading response	-0.16±0.07	-0.19±0.09	**-0.27±0.19ᶲ**	-0.30±0.24	**-0.48±0.26ᵡᶲ**
Power Abs—Terminal stance	-0.62±0.32	**-0.61±0.47ᶲ**	-0.82±0.62	**-1.21±0.53ᵡᶲ**	-1.02±0.60
Power Gen—Preswing	2.98±0.69	**4.15±1.61ᶲ**	**4.38±1.19ᵡ**	**1.87±0.63ᵡᶲ**	**3.79±2.01ᵑ**
**Knee Moments (Nm/kg) & Powers (W/kg)**					
Flexor Mom—Loading response	-0.53±0.11	**-0.59±0.17ᶲ**	**-0.78±0.25ᵡᶲᵑ**	**-0.37±0.13ᵡᶲ**	**-0.56±0.23ᶲᵑ**
Extensor Mom—Midstance	0.58±0.34	**0.85±0.30ᶲ**	**0.53±0.16ᶲᵑ**	**0.13±0.23ᵡᶲ**	**0.05±0.18ᵡᶲ**
Flexor Mom—Terminal stance	-0.47±0.11	**-0.57±0.19ᶲ**	**-0.78±0.33ᶲ**	**-0.41±0.23ᶲ**	**-0.58±0.30ᶲᵑ**
Power Abs—Loading response	-0.38±0.41	**-0.80±0.57ᶲ**	-0.57±0.38	**-0.23±0.10ᶲ**	-0.39±0.33
Power Gen—Midstance	0.58±0.44	**1.03±0.46ᶲ**	**0.57±0.25ᶲᵑ**	**0.13±0.16ᵡᶲ**	**0.13±0.11ᵡᶲ**
Power Gen—Terminal stance	0.77±0.42	**0.76±0.34ᶲ**	**1.07±0.55ᶲ**	**0.46±0.29ᶲ**	**0.67±0.37ᶲᵑ**
**Hip Moments (Nm/kg) & Powers (W/kg)**					
Extensor Mom—Loading response	1.11±0.25	**1.51±0.47ᶲ**	**1.66±0.46ᵡᶲ**	**1.06±0.31ᶲ**	**1.28±0.40ᶲ**
Flexor Mom—Terminal stance	-0.64±0.20	**-0.49±0.12ᶲ**	**-0.60±0.28ᶲ**	**-0.72±0.27ᶲ**	**-0.92±0.52ᶲ**
Extensor Mom—Swing	0.42±0.08	**0.52±0.10ᶲ**	**0.54±0.12ᶲ**	**0.43±0.10ᶲ**	**0.32±0.10ᶲᵑ**
Power Gen—Midstance	1.12±0.28	1.68±0.77	1.58±0.64	**1.93±0.85ᵡ**	**1.98±0.57ᵡ**
Power Abs—Terminal stance	-0.52±0.23	-0.30±0.16	-0.45±0.32	-0.48±0.34	-0.64±0.48
Power Gen—Terminal stance	0.79±0.11	0.90±0.25	**0.95±0.32ᶲ**	1.04±0.26	**1.29±0.40ᵡᶲᵑ**
**Ground Reaction Forces (Body weight normalized)**					
First Vertical Peak	1.02±0.08	**1.12±0.18ᶲ**	1.17±0.30	**0.98±0.16ᶲ**	1.14±0.34
Vertical Minimum—Midstance	0.74±0.04	**0.58±0.14ᵡᶲ**	0.74±0.26	**0.65±0.13ᶲ**	0.75±0.21
Second Vertical Peak	1.11±0.06	**1.13±0.22ᶲ**	**1.31±0.36ᶲ**	**1.01±0.19ᶲ**	**1.19±0.39ᶲ**
Braking	-0.15±0.04	-0.17±0.04	-0.18±0.07	-0.14±0.04	**-0.20±0.07ᵑ**
Propulsion	0.17±0.02	**0.23±0.06ᵡᶲ**	**0.26±0.08ᵡᶲ**	**0.13±0.03ᵡᶲ**	**0.17±0.08ᶲ**

Note: Data are mean ± SD

Abbreviations: Absorption (Abs), Generation (Gen), Moment (Mom)

**Bold:** Significant values

**ᵡ** Significantly different from the AB limb (p < 0.0125)

**ᶲ** Significantly different from the contralateral limb of the same prosthetic condition (p < 0.05)

**ᵑ** Significantly different from the same limb of the ESR condition (p < 0.05)

**Fig 2:**
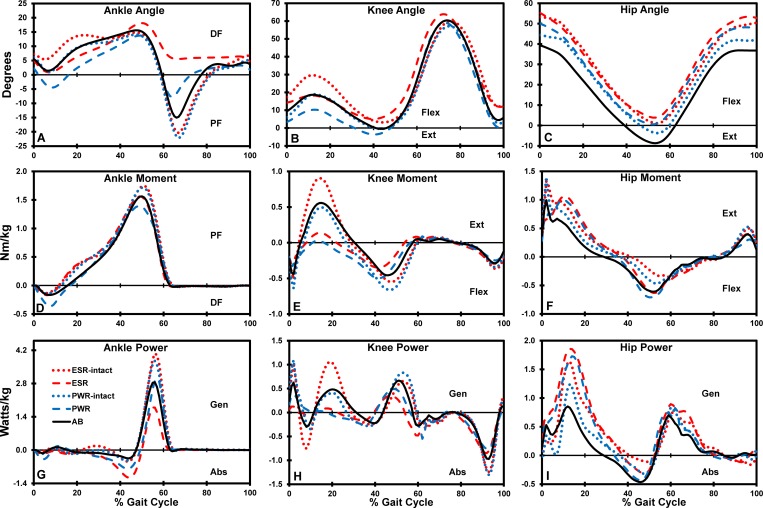
Group average sagittal plane ankle, knee, and hip kinematics and kinetics. Data shown while walking at a controlled velocity up a 5° slope, normalized to gait cycle. Lines represent means for AB group, ESR limb, ESR intact limb, PWR limb, and PWR intact limb. Abbreviations: Absorption (Abs), Extensor (Ext), Flexor (Flex), Dorsiflexion (DF), Generation (Gen), Plantarflexion (PF).

When transitioning off the prosthetic limb, peak plantarflexion at initial swing in the prosthetic limb was significantly less (p < 0.001) with both devices compared to the AB limb. Peak plantarflexion in the prosthetic limb significantly increased (p < 0.001) approximately 13° with the PWR compared to the ESR (p < 0.001, Fig 2A). The leading intact limb during the ESR condition demonstrated significantly less (p < 0.009) peak plantarflexion (more dorsiflexion) at loading response compared to PWR condition.

### Knee Angle

Prosthetic limb peak knee flexion in loading response was significantly less than the intact limb during both prosthetic conditions (ESR: 37% and PWR: 43%; p < 0.005; Table 3A). Prosthetic limb peak knee flexion in swing was significantly greater than the intact limb during both prosthetic conditions (ESR: 7% and PWR: 6%; p < 0.045; Table 3A).

When transitioning onto the prosthetic limb, peak knee flexion at initial contact of the PWR limb was approximately 11° less than the ESR limb (p < 0.002). During loading response the PWR prosthetic limb peak knee flexion was approximately 8° less than AB and ESR prosthetic limb values (p < 0.028; Fig 2B).

When transitioning off the prosthetic limb, peak knee flexion at loading response of the leading intact limb was approximately 10° greater with the ESR compared to the AB limb (p < 0.001). During PWR use, peak knee flexion at loading response of the intact limb decreased by approximately 11° compared to the ESR condition (p < 0.004). At initial contact with the PWR, peak knee flexion of the intact limb was approximately 11° less than when using the ESR (p < 0.001).

### Hip and Pelvis Angle

Participants in the TTA group exhibited greater peak hip flexion in their prosthetic and intact limbs for both prosthetic conditions throughout gait compared to the AB group (2–16°; p < 0.012; Fig 2C). The TTA group exhibited greater maximum anterior pelvic tilt than the AB group (p < 0.004) regardless of device which may have contributed the overall increase in hip flexion observed in the TTA group. However, there was no significant difference in maximum anterior pelvic tilt between devices. Further, with the PWR, hip flexion peaks in the prosthetic limb were significantly greater than the intact limb throughout gait (4–7°; p < 0.014) and not likely related to pelvic inclination.

When transitioning onto the prosthetic limb, peak hip flexion at loading response of the ESR limb was approximately 16° greater (p < 0.001) than the AB limb and decreased with the PWR (p < 0.020). Peak hip extension at preswing of trailing intact limb was significantly less (p < 0.001) with the ESR compared to the AB limb and increased toward AB values with the PWR (p < 0.029).

When transitioning off the prosthetic limb, peak hip extension at preswing of the prosthetic limb was approximately 12° less with the ESR (p < 0.003) and 9° less with the PWR (p < 0.006) compared to the AB limb. Peak hip flexion at loading response of the leading intact limb with the ESR was approximately 15° greater (p < 0.001) than the AB limb and significantly decreased with the PWR (p < 0.048).

### Ankle Moment and Power

When transitioning onto the prosthetic limb, the peak dorsiflexor moment at loading response was significantly greater in the PWR limb compared to the AB and ESR limbs (p < 0.025; Fig 2D, Table 3B). Similarly, the peak ankle power absorption at loading response was significantly greater with the PWR compared to the AB limb (p < 0.001; Fig 2G). With the ESR, peak plantarflexion power generation in the trailing intact ankle at preswing was approximately 39% greater than the AB limb, but the change was not significant (p = 0.056). In contrast, peak plantarflexion power generation in the intact ankle at preswing with the PWR was significant greater than the AB limb (47%; p = 0.005).

When transitioning off the prosthetic limb, peak plantarflexion power generation at preswing in the ESR limb was approximately 37% less than the AB limb (p < 0.0125) and approximately 55% less than the intact limb ankle (p < 0.001). Peak plantarflexion power generation at preswing in the PWR limb was approximately 103% greater than the ESR limb (p = 0.018) and not significantly different from the AB and intact limbs. There were no significant differences in intact leading limb peak ankle moments and powers at loading response when compared to the AB limb.

### Knee Moment and Power

In general, the peak knee extensor moment and power generation during stance with either device were significantly less in the prosthetic limb compared to the intact limb (p < 0.007; Fig 2E and 2H).

When transitioning onto the prosthetic limb, the peak knee extensor moment in the prosthetic limb with either device was significantly lower than the AB limb (p < 0.003; Table 3B). Similarly, peak knee power generation during midstance was significantly reduced in the prosthetic limb compared to AB values (p < 0.009) with both devices (Fig 2H). There were no significant differences in intact trailing limb peak knee moments and powers at terminal stance with either device when compared to the AB limb.

When transitioning off the prosthetic limb, there were no significant differences in the peak prosthetic limb moments and powers at terminal stance with either device when compared to the AB limb. During PWR use, the peak knee flexor moment in the prosthetic limb at terminal stance was significantly greater than with the ESR (p = 0.040). Peak knee power generation in the prosthetic limb at terminal stance with the PWR approached AB values and was significantly greater than with the ESR (p = 0.036). The peak knee extensor moment and power generation in the leading intact limb at midstance with the ESR was approximately 47% and 78% greater than AB values, yet not significant (p = 0.038). The peak knee extensor moment and power generation in the leading intact limb at midstance was significantly reduced with the PWR compared to the ESR (p < 0.006) and normalized toward AB values.

### Hip Moment and Power

In general, the peak hip extensor moments in loading response and swing with either device were significantly less in the prosthetic limb compared to the intact limb (p < 0.019; Fig 2F and 2I). The peak hip flexor moments in terminal stance with either device were significantly greater in the prosthetic limb compared to the intact limb (p < 0.025; Fig 2F and 2I).

When transitioning onto the prosthetic limb, the peak hip-power generations in midstance with either device were significantly greater in the prosthetic limb compared to the AB limb (ESR: 72% and PWR: 77%; p < 0.011; Table 3B). There were no significant differences in intact trailing limb peak hip moments and powers at terminal stance with either device when compared to the AB limb.

When transitioning off the prosthetic limb, peak power generations at terminal stance of the prosthetic limb were greater than AB values with both devices, but only significant with the PWR (p = 0.003). Further, peak power generation at terminal stance of the PWR limb was significantly greater than the ESR limb (p = 0.034) and the intact limb (p = 0.016). The peak hip extensor moments in loading response of the leading intact limb were greater than AB values with both devices, but only significant with the PWR (p = 0.004).

### Ground Reaction Forces

During ESR use, the first peak vertical force was significantly less (13%, p = 0.001) in the prosthetic limb than the intact limb (Table 3B). This asymmetry during loading response was reversed at midstance when the ESR limb exhibited significantly greater (12%, p = 0.001) vertical force minimum compared to the intact limb. In addition, the intact limb vertical force minimum at midstance when using the ESR was significantly less (22%, p = 0.006) than in the AB group. In contrast, no significant differences in vertical force peaks were observed between limbs when transitioning onto the PWR limb; thus, demonstrating more symmetric limb loading than with the ESR. The three vertical ground reaction force values measured when using the PWR were not significantly different from ESR and AB values, for either limb. When transitioning onto the prosthetic limb, peak braking force in the PWR limb was significantly greater (43%, p = 0.01) than in the ESR limb.

The second vertical force peak was significantly less in the prosthetic limb than the intact limb with either device (ESR: 11% and PWR: 9%; p < 0.005). Similarly, the peak propulsive force at push-off was significantly smaller in the prosthetic limb than in the intact limb with either device (ESR: 43% and PWR: 35%; p < 0.001). The peak propulsive force at push-off was significantly greater in the intact limb compared to the AB limb with either device (ESR: 35% and PWR: 53%; p < 0.006). In contrast, the peak propulsive force at push-off was significantly less in the ESR limb than the AB limb (24%, p = 0.002). With the PWR, prosthetic limb peak propulsive force at push-off approximated the AB limb value; however, was not significantly greater than the ESR limb.

## Discussion

**Fig 3:**
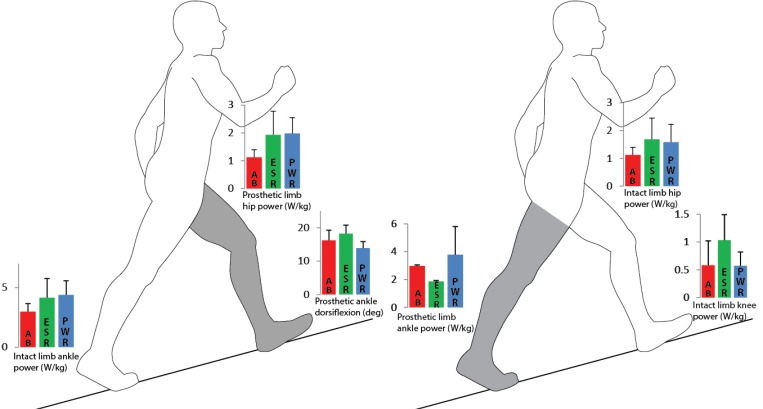
Graphic representation of the transition onto the shaded prosthetic limb (Left) and the transition off the shaded prosthetic limb (Right). Graph inserts on Left depict AB, ESR, and PWR peak power generation values at the prosthetic limb hip and intact limb ankle, and peak dorsiflexion of the prosthetic ankle. Graph inserts on Right depict AB, ESR, and PWR peak power generation values at the intact limb hip, knee, and prosthetic ankle. See Table 3B for exact values and statistical significance. Transitioning onto the prosthetic limb was assessed from initial contact through midstance. Transitioning off the prosthetic limb was assessed from terminal stance through preswing.

In this study, the PWR demonstrated significant increases in prosthetic ankle ROM and peak plantarflexion power generation compared to the ESR, and more closely matched AB values. When transitioning onto the leading prosthetic limb, the TTA group exhibited similar compensations with the PWR at the intact limb ankle and prosthetic limb hip as also observed with the ESR (Fig 3, Left). In contrast, when transitioning off the trailing prosthetic limb, increased ankle power and ROM provided by the PWR contributed to decreased intact limb knee extensor power generation relative to the ESR, lessening demand on the intact limb knee (Fig 3, Right).

### Temporal Spatial

Previous studies [6–8] have reported significantly slower self-selected speeds in individuals with TTA walking up slopes compared to AB individuals performing the same task. Findings from these reports suggest that individuals with TTA may have walked slower due to reduced functional status compared to the AB groups. In contrast to these reports and our hypothesis, the TTA group, while using either device, self-selected walking velocities faster than the AB group (ESR: 12%, PWR: 18%). Compared to ESR use, PWR use did not significantly change the self-selected walking velocities of the TTA group. Our results likely differ from previous reports due to differences in the pre-amputation functional ability and health status of the participants. Specifically, the TTA group in our study was comprised of young, highly fit service members with traumatic amputations who had undergone intensive rehabilitation following their injuries. In contrast, participants in previous reports were generally older (e.g., mean age: 50 years [6, 8] and 71 years [7]) with some having undergone amputation due to dysvascular disease.

To account for the potentially confounding effect of walking velocity on slope ascent mechanics [7], we calculated a controlled velocity scaled to each participant using leg length and a Froude number of 0.16 [17]. In this data set, there was no statistical difference between the controlled and self-selected walking velocities. Data collected at self-selected walking velocity resulted in similar statistical differences between groups and devices (see S1 and S2 Tables and S1 Fig).

When using the ESR, the TTA group spent significantly less time in prosthetic limb stance compared to their intact limb. This between limbs difference in stance time was similar to a previous report of level ground gait with an ESR [10]; however, the difference was small (0.03s) and may not have resulted in a clinically meaningful disruption of gait mechanics. In addition, a small (0.03s) non-significant increase in prosthetic limb stance time was exhibited with the PWR compared to ESR use. This resulted in no significant between limbs difference in stance time with the PWR. The increase in prosthetic limb stance time with the PWR was similar to findings in level ground gait [10]. There are many factors that may have influenced this increase in stance time. One is the mechanical difference between ESR and PWR devices which effect prosthetic ankle ROM and the duration of prosthetic foot contact with the floor near toe-off. The ESR devices were comprised of a passive foot that deforms during loading and only returns to a neutral position when unloaded. In contrast, the PWR used a motor in conjunction with a deformable foot resulting in an additional 13° of plantarflexion. This increase in plantarflexion with the PWR likely prolonged the time the PWR was in contact with the floor compared to the ESR. While this increase in prosthetic limb stance time with the PWR was small, the underlying increase in plantarflexion ROM appeared to be beneficial across different walking environments [9–11, 13].

### Transitioning onto the Prosthetic Limb

When AB individuals walk up slopes, they position the leading limb with increased ankle dorsiflexion, knee flexion, and hip flexion at initial contact compared to level ground walking [2]. This facilitates placement of the leading limb on the slope’s elevated surface. In contrast, individuals with TTA walking up slopes appeared to have difficulties transitioning onto to their prosthetic limb due to limited prosthetic limb ankle dorsiflexion [6, 7]. In agreement with our hypothesis, reduced peak prosthetic ankle dorsiflexion with both devices resulted in compensations at the prosthetic limb knee and hip when the TTA group transitioned onto the leading prosthetic limb (Fig 3, Left).

Consistent with previous studies [6, 7], when using the ESR, the TTA group exhibited significant reductions in the prosthetic limb knee extensor moment and power generation peaks at midstance compared to the AB limb. When using the PWR, the prosthetic limb knee extensor moment and power generation peaks at midstance further decreased coinciding with a significant reduction in prosthetic limb stance knee flexion relative to the ESR and AB limbs. We observed less peak dorsiflexion in the PWR limb compared to the ESR limb, likely limiting the ability of the prosthetic limb shank to rotate over the PWR and move the knee anteriorly. A prosthetic ankle-foot’s ability to dorsiflex when transitioning onto the prosthetic limb was associated with greater prosthetic limb stance knee flexion and extensor moments [6]. Therefore, had the PWR been able to actively dorsiflex facilitating anterior movement of the prosthetic limb knee, deficits in knee extensor moment and power generation peaks may have been improved. While aligning the PWR in more dorsiflexion may have resulted in improved prosthetic knee kinetics during slope ascent, this could have detrimental effects on level ground. Instead, an adaptive system for active prosthetic ankle dorsiflexion [6] may be a beneficial feature to add in future PWR designs.

As hypothesized when transitioning onto the prosthetic limb, the TTA group exhibited greater intact limb ankle power generation peaks relative to AB values while using either device (Fig 3, ESR: 39%, PWR: 47%). This was evident by greater intact limb propulsive ground reaction forces compared to AB and prosthetic limb values. Power generated by trailing limb plantarflexors at push-off can best restore energy lost when transitioning to the leading limb [9, 22, 23]. In this current study, trailing intact limb plantarflexors at push-off appeared to compensate for reduced prosthetic limb dorsiflexion and the associated reduction in prosthetic limb knee extensor power generation at midstance. Additionally, prosthetic limb hip extensors appeared to aid the intact limb plantarflexors in propelling the body up the slope. This was demonstrated by a greater hip extensor power generation peak in the prosthetic limb with both devices during midstance when compared to the AB limb.

Compensations observed here were in contrast to previous studies reporting reduced hip moments and powers when walking up slopes [6–8] and suggest that individuals with TTA are less able to use the hip for propulsion [6]. The differences between our findings and those in the literature may also be due to the differences in TTA group walking speeds. In studies by Vickers [7], Vrieling [8], and Fradet [6], general reductions of all moments and powers were observed bilaterally in the TTA groups and reported to contribute to slower walking velocities compared to the AB groups [6–8]. In our study, the TTA and AB groups walked at a speed normalized to leg length to control for the influence of speed on gait mechanics. By requiring TTA and AB groups to walk at controlled velocities, dissimilarities in gait mechanics can be attributed to functional differences between groups and/or devices rather than walking velocity.

### Transitioning off the Prosthetic Limb

When walking up slopes and transitioning off the trailing limb, AB individuals must increase extension though the trailing limb in order to elevate and propel the body’s center of mass uphill [2]. Trailing limb ankle plantarflexion at push-off is a key contributor to step-to-step transition work needed for forward and upward propulsion [9]. Limited ROM and push-off power in the trailing limb requires the leading limb to compensate if the body is to be propelled uphill. For example, individuals with TTA using passive prosthetic ankle-feet exhibited compensations in the intact leading limb due to limited ankle ROM and power generation in the trailing prosthetic limb [6–9].

Consistent with previous reports [6–8], when transitioning off the trailing ESR limb the TTA group exhibited greater leading intact limb hip and knee flexion at loading response compared to the AB limb. This served to shorten the intact limb to compensate for a functionally shorter prosthetic limb due to approximately 20° less ESR plantarflexion relative to the AB limb. Greater intact limb hip and knee flexion at loading response may have also contributed to the reduction in vertical ground reaction force minimum observed in the intact limb at midstance.

As hypothesized and previously reported [10], peak ankle power generation in the ESR limb at preswing was 37% and 55% less than AB and intact limb values, respectively (Fig 3). This partially contributed to a 24% and 43% decrease in prosthetic limb propulsion when transitioning off the ESR as compared to AB and intact limb values, respectively. While not significant, when using the ESR, intact limb knee and hip extensor moments and power generation peaks were 36–78% greater than the AB limb in early stance. Given the lack of trailing prosthetic limb ankle power generation with the ESR, an increase in intact limb knee and hip extensor power generation in early stance would be necessary to help transition the body uphill onto the leading intact limb (Fig 3, Right).

Similar to previous reports [9, 10], we observed greater peak ankle plantarflexion (13°) and power generation (103%) at preswing in the PWR limb compared to the ESR limb. As hypothesized, with PWR use compensations exhibited at the intact limb knee were reduced. We observed a reduction in intact limb knee flexion at loading response while using the PWR. This suggested that the trailing PWR limb was functionally longer than with ESR use, thus requiring less compensation by the leading intact limb. The reduction in intact limb knee flexion at loading response with the PWR coincided with a nonsignificant increase in the intact limb vertical ground reaction force peak at midstance as compared to ESR use, and matched the AB limb value. In addition, with the PWR, intact limb knee extensor moment and power generation peaks decreased relative to ESR use. This resulted in intact limb knee extensor moment and power generation peaks that were closer to AB values (Fig 3, Right). The increase in prosthetic limb ankle power generation at push-off with the PWR appeared to have decreased demand on the intact limb knee. Contrary to our hypothesis, increased plantarflexion and push-off power by the PWR did not reduce the intact limb hip extensor moment or power generation in early stance. In fact, the peak hip extensor moment of the intact limb at loading response became significantly greater than the AB limb with PWR use.

Active power generation by the PWR appeared to increase propulsive force at push-off in the PWR limb to match the AB limb; however, the peak value was not significantly greater than the ESR limb. Further, peak propulsive force at push-off in the PWR limb was 35% less than the intact limb. This suggests that although peak push-off power in prosthetic limb was greater than with the ESR, it was not effective in fully matching the peak propulsive force of the intact limb. One potential reason for the persistent deficit is the uniarticular action of the device as compared to the biarticular gastrocnemius [24]. It has been previously suggested that power provided by a uniarticular ESR [25] and PWR [10] is underutilized for forward propulsion and results in increased prosthetic limb hip power generation during level ground gait. A study by Lewis and colleagues [26] demonstrated that increased ankle push-off power resulted in a decreased peak hip extensor moment during level ground walking. In contrast and despite the push-off power provided by the PWR, the PWR limb exhibited greater hip extensor power generation during terminal stance than the AB, intact, and ESR limbs. This likely helped to compensate for the underutilized push-off power generated by the PWR.

### Limitations

An individual’s activity level prior to amputation and the mechanism of the amputation can influence post-amputation outcomes such as gait quality [27]. For example, the TTA cohort in this study was comprised of highly active and fit service members with relatively recent traumatic amputations. Thus, these results may not be representative of the general population of individuals with limb loss. In current slope walking literature, TTA populations are generally comprised of older individuals (>50 years) with dysvascular amputations [7, 8] which may respond differently to the use of a powered ankle-foot prosthesis. In addition, the findings presented here may not generalize to all slope angles a person may encounter.

## Conclusions

While the PWR provided active ankle plantarflexion and push-off power when transitioning off the prosthetic limb, it was not capable of active dorsiflexion. Thus, the PWR functioned similar to a passive ESR device during the transition onto the prosthetic limb resulting in similar prosthetic limb hip and intact limb ankle compensations. In contrast, when transitioning off the prosthetic limb, the increased ankle plantarflexion and push-off power provided by the PWR contributed to decreased intact limb knee extensor power production, lessening demand on the intact limb knee. Further work is needed to determine whether the provided active ankle plantarflexion and push-off power would improve slope descent gait mechanics.

## Supporting Information

S1 Fig Group average sagittal plane ankle, knee, and hip kinematics and kinetics while walking at self-selected velocity up a 5° slope, normalized to gait cycle. Lines represent means for AB group, ESR limb, ESR intact limb, PWR limb, and PWR intact limb. Abbreviations: Absorption (Abs), Extensor (Ext), Flexor (Flex), Generation (Gen).
(TIFF)

S1 Table Temporal-spatial measures at a self-selected velocity walking up a 5° slope. Measures are shown for the right limb of the able-bodied group and the contralateral intact and prosthetic limbs of the TTA group. The controlled and self-selected walking velocities are also shown.
(DOCX)

S2 Table Peak joint angles, moments, and powers for the ankle, knee, and hip at a self-selected velocity walking up a 5° slope. Measures are shown for the right limb of the able-bodied group and the intact and prosthetic limbs of the TTA group.
(DOCX)
