# Supplementary figures and images for: Evaluation of a Powered Ankle-Foot Prosthesis during Slope Ascent Gait
Source: PLoS One. 2016 Dec 15;11(12):e0166815. doi: 10.1371/journal.pone.0166815 (PMC5157979; doi:10.1371/journal.pone.0166815)

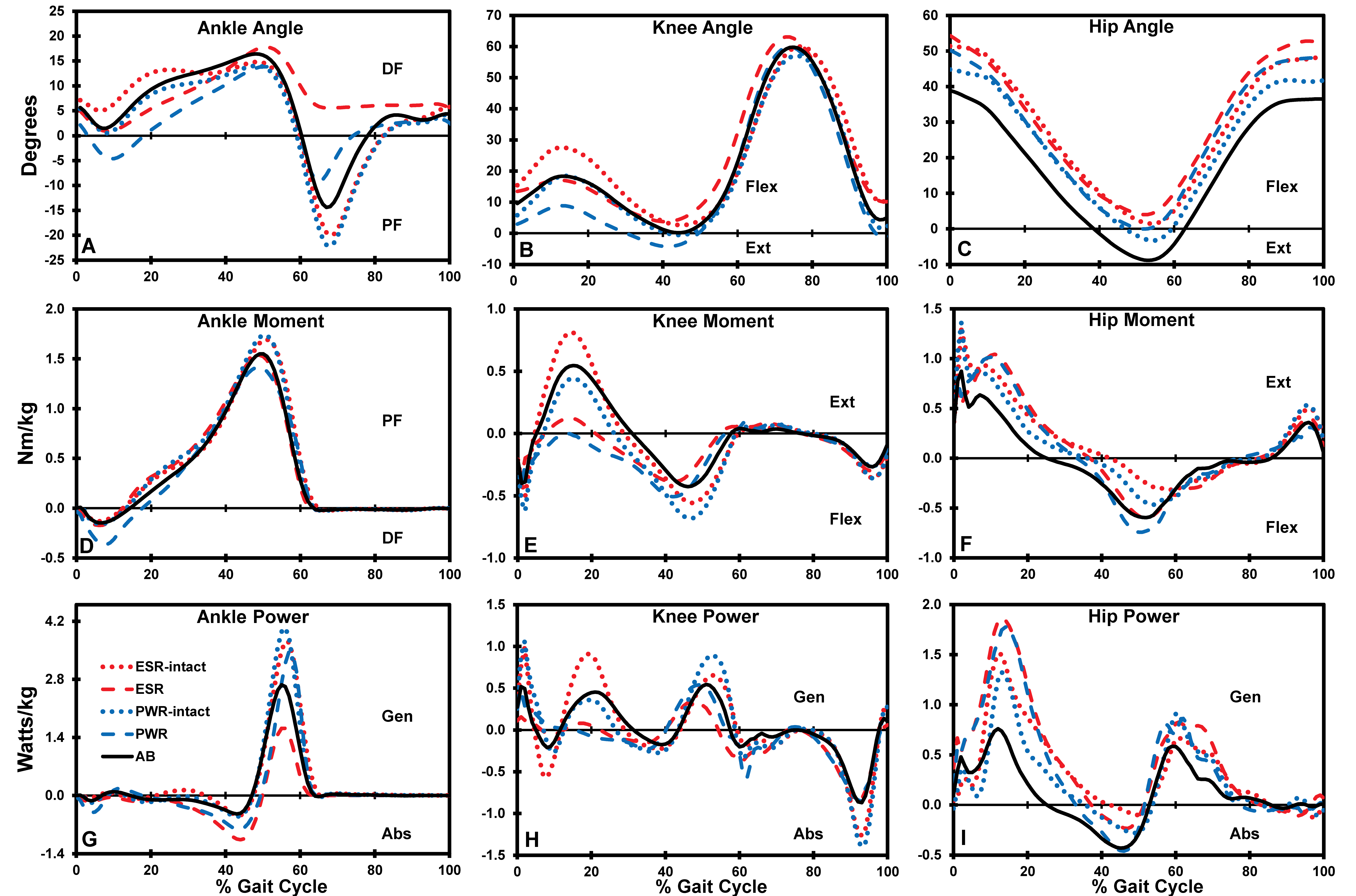

Supplement: S1 Fig — Lines represent means for AB group, ESR limb, ESR intact limb, PWR limb, and PWR intact limb. Abbreviations: Absorption (Abs), Extensor (Ext), Flexor (Flex), Generation (Gen). (TIFF) [file pone.0166815.s001.tiff]
