# Supplementary material for: Evaluation of a Powered Ankle-Foot Prosthesis during Slope Ascent Gait
Source: PLoS One. 2016 Dec 15;11(12):e0166815. doi: 10.1371/journal.pone.0166815 (PMC5157979; doi:10.1371/journal.pone.0166815)
Supplement: S1 Table — Measures are shown for the right limb of the able-bodied group and the contralateral intact and prosthetic limbs of the TTA group. The controlled and self-selected walking velocities are also shown. (DOCX) [file pone.0166815.s002.docx]

| **Measures** | **Able-Bodied** | **TTA Intact Limb** | | **TTA Prosthetic Limb** | |
| --- | --- | --- | --- | --- | --- |
|  |  | **ESR** | **PWR** | **ESR** | **PWR** |
| Stance Time (s) | 0.77±0.07 | **0.75±0.05ᶲ** | 0.73±0.05 | **0.71±0.04ᶲ** | 0.73±0.03 |
| Step Length (m) | 0.73±0.05 | **0.73±0.06ᶲ** | **0.76±0.09ᶲ** | **0.81±0.05ᵡᶲ** | **0.85±0.08ᵡᶲ** |
| Step Time (s) | 0.62±0.05 | 0.58±0.04 | **0.57±0.03ᶲ** | 0.60±0.04 | **0.59±0.03ᶲ** |
| Stride Length (m) | 1.46±0.10 | 1.54±0.10 | 1.59±0.16 | 1.55±0.10 | 1.62±0.18 |
| Swing Time (s) | 0.46±0.02 | **0.43±0.03ᵡᶲ** | **0.43±0.03ᵡᶲ** | **0.46±0.03ᶲ** | **0.44±0.02ᶲᵑ** |
| Controlled Velocity (m/s) | 1.23±0.07 | **1.36±0.07ᵡ** | **1.36±0.11ᵡ** | **1.36±0.07ᵡ** | **1.36±0.11ᵡ** |
| Self-Selected Velocity (m/s) | 1.18±0.06 | **1.32±0.10ᵡ** | **1.39±0.18ᵡ** | **1.32±0.10ᵡ** | **1.39±0.18ᵡ** |

Note: Data are mean ± SD

**Bold:** Significant values

**ᵡ** Significantly different from the AB limb (p < 0.0125)

**ᶲ** Significantly different from the contralateral limb of the same prosthetic condition (p < 0.05)

**ᵑ** Significantly different from the same limb of the ESR condition (p < 0.05)
