# Supplementary material for: Evaluation of a Powered Ankle-Foot Prosthesis during Slope Ascent Gait
Source: PLoS One. 2016 Dec 15;11(12):e0166815. doi: 10.1371/journal.pone.0166815 (PMC5157979; doi:10.1371/journal.pone.0166815)
Supplement: S2 Table — Measures are shown for the right limb of the able-bodied group and the intact and prosthetic limbs of the TTA group. (DOCX) [file pone.0166815.s003.docx]

| **(A) Kinematic Parameters Peaks** | **Able-Bodied** | **TTA Intact Limb** | | **TTA Prosthetic Limb** | |
| --- | --- | --- | --- | --- | --- |
|  |  | **ESR** | **PWR** | **ESR** | **PWR** |
| **Ankle angle (°)** |  |  |  |  |  |
| Plantarflexion - Loading response | 1.10±3.11 | **4.16±4.25ᶲ** | **0.14±3.58ᶲᵑ** | **0.64±4.03ᶲ** | **-4.81±3.26ᵡᶲᵑ** |
| Dorsiflexion - Terminal stance | 16.92±3.05 | 16.54±2.69 | 15.27±2.40 | 18.13±2.75 | **14.04±1.93ᵑ** |
| Plantarflexion - Initial swing | -15.22±3.00 | **-21.21±4.54ᵡ** | **-22.57±5.41ᵡᶲ** | **4.89±2.92ᵡᶲ** | **-9.86±3.39ᵡᶲᵑ** |
| Sagittal range of motion | 32.14±4.67 | 37.75±5.20 | **37.84±5.23ᶲ** | **17.49±4.12ᵡᶲ** | **23.91±4.13ᵡᶲᵑ** |
| **Knee angle (°)** |  |  |  |  |  |
| Flexion - Initial contact | 9.52±6.78 | 15.43±9.58 | **5.61±6.16ᵑ** | 13.42±8.94 | **3.06±5.20ᵑ** |
| Flexion - Loading response | 18.82±5.25 | **27.81±7.42ᵡᶲ** | **18.69±5.66ᶲᵑ** | **17.82±9.22ᶲ** | **9.68±5.35ᵡᶲᵑ** |
| Extension - Terminal stance | -0.38±3.51 | 2.14±4.52 | -1.86±5.34 | 2.97±7.67 | **-3.76±5.70ᵑ** |
| Flexion - Swing | 60.32±3.08 | 60.32±6.76 | **57.02±5.32ᶲ** | 63.67±3.11 | **60.82±4.00ᶲ** |
| Sagittal range of motion | 61.77±3.03 | 59.01±6.24 | **61.30±4.28ᶲ** | 61.09±7.60 | **65.89±6.69ᶲᵑ** |
| **Hip angle (°)** |  |  |  |  |  |
| Flexion - Loading response | 38.90±4.86 | **51.85±8.51ᵡ** | **45.21±6.30ᶲ** | **54.24±8.46ᵡ** | **50.18±5.84ᵡᶲ** |
| Extension - Preswing | -9.14±5.65 | **1.14±6.68ᵡ** | **-3.45±6.11ᶲᵑ** | **3.33±9.11ᵡ** | **-0.32±7.13ᵡᶲ** |
| Flexion - Swing | 37.42±3.53 | **48.94±8.94ᵡ** | **42.73±7.20ᶲ** | **53.39±6.78ᵡ** | **49.37±5.73ᵡᶲ** |
| Sagittal range of motion | 48.53±4.75 | 50.94±6.25 | 49.34±7.10 | 51.96±4.87 | 51.61±7.46 |
| **Pelvis angle (°)** |  |  |  |  |  |
| Max Anterior tilt | 11.56±4.50 | **21.25±7.94ᵡ** | **19.39±4.05ᵡᶲ** | **21.65±7.47ᵡ** | **20.88±4.25ᵡᶲ** |
| Min Anterior tilt | 7.64±4.62 | **15.93±7.49ᵡ** | **14.41±4.15ᵡᶲ** | **16.14±7.50ᵡ** | **15.44±3.94ᵡᶲ** |
| Sagittal range of motion | 3.92±1.09 | 5.32±1.74 | 4.98±1.85 | 5.51±2.30 | 5.44±2.10 |
| **(B) Kinetic Parameter Peaks** |  |  |  |  |  |
|  |  |  |  |  |  |
| **Ankle Moments (Nm/kg) & Powers (W/kg)** |  |  |  |  |  |
| Dorsiflexion Mom - Loading response | -0.16±0.05 | **-0.15±0.07ᶲ** | **-0.20±0.12ᶲ** | **-0.23±0.13ᶲ** | **-0.44±0.23ᵡᶲᵑ** |
| Plantarflexion Mom - Terminal stance | 1.58±0.15 | **1.64±0.33ᶲ** | **1.93±0.58ᶲ** | **1.48±0.32ᶲ** | **1.58±0.56ᶲ** |
| Power Abs - Loading response | -0.15±0.07 | -0.15±0.07 | **-0.27±0.20ᶲ** | -0.28±0.21 | **-0.51±0.34ᵡᶲ** |
| Power Abs - Terminal stance | -0.66±0.32 | **-0.61±0.44ᶲ** | -0.82±0.65 | **-1.15±0.46ᵡᶲ** | -1.07±0.75 |
| Power Gen - Preswing | 2.87±0.83 | 3.87±1.36 | **4.54±1.34ᵡ** | **1.78±0.56ᵡᶲ** | **4.23±2.08ᵑ** |
| **Knee Moments (Nm/kg) & Powers (W/kg)** |  |  |  |  |  |
| Flexor Mom - Loading response | -0.46±0.13 | **-0.57±0.19ᶲ** | **-0.80±0.37ᶲᵑ** | **-0.35±0.15ᶲ** | **-0.58±0.31ᶲᵑ** |
| Extensor Mom - Midstance | 0.56±0.30 | 0.75±0.17 | **0.50±0.14ᶲᵑ** | **0.10±0.19ᵡᶲ** | **0.03±0.17ᵡᶲ** |
| Flexor Mom - Terminal stance | -0.45±0.13 | **-0.57±0.17ᶲ** | **-0.79±0.33ᵡᶲ** | **-0.43±0.21ᶲ** | **-0.58±0.35ᶲ** |
| Power Abs - Loading response | 0.62±0.31 | **1.16±0.53ᶲ** | **1.47±1.09ᶲ** | **0.29±0.20ᵡᶲ** | **0.85±0.91ᶲ** |
| Power Gen - Midstance | -0.33±0.28 | **-0.61±0.33ᶲ** | -0.47±0.27 | **-0.20±0.12ᶲ** | -0.38±0.37 |
| Power Gen - Terminal stance | 0.76±0.30 | **1.00±0.25ᶲ** | **1.13±0.62ᶲ** | **0.50±0.23ᶲ** | **0.75±0.48ᶲ** |
| **Hip Moments (Nm/kg) & Powers (W/kg)** |  |  |  |  |  |
| Extensor Mom - Loading response | 0.97±0.18 | **1.40±0.47ᶲ** | **1.70±0.71ᵡᶲ** | **1.04±0.26ᶲ** | **1.31±0.59ᶲ** |
| Flexor Mom - Terminal stance | -0.63±0.17 | **-0.44±0.14ᵡᶲ** | **-0.61±0.31ᶲ** | **-0.70±0.26ᶲ** | **-0.96±0.56ᶲ** |
| Extensor Mom - Swing | 0.37±0.08 | **0.52±0.13ᵡ** | **0.55±0.13ᵡᶲ** | 0.43±0.14 | **0.35±0.11ᶲᵑ** |
| Power Gen - Midstance | 0.99±0.16 | **1.54±0.69ᶲ** | 1.71±0.90 | **1.94±0.61ᵡᶲ** | **2.10±0.84ᵡ** |
| Power Abs - Terminal stance | -0.49±0.21 | -0.27±0.18 | **-0.45±0.42ᶲ** | -0.44±0.30 | **-0.63±0.41ᶲ** |
| Power Gen - Terminal stance | 0.72±0.14 | **0.84±0.24ᶲ** | **0.96±0.50ᶲ** | **1.01±0.27ᵡᶲ** | **1.43±0.63ᵡᶲᵑ** |
| **Ground Reaction Forces (Body weight normalized)** |  |  |  |  |  |
| First Vertical Peak | 1.01±0.05 | 1.08±0.14 | 1.15±0.34 | **0.97±0.14ᶲ** | 1.14±0.33 |
| Vertical Minimum - Midstance | 0.76±0.05 | **0.62±0.15ᵡᶲ** | 0.73±0.20 | **0.66±0.15ᶲ** | 0.74±0.20 |
| Second Vertical Peak | 1.11±0.07 | **1.11±0.19ᶲ** | **1.31±0.38ᶲ** | **1.01±0.18ᶲ** | **1.21±0.44ᶲ** |
| Braking | -0.15±0.03 | -0.15±0.03 | -0.18±0.07 | -0.13±0.03 | **-0.19±0.07ᵑ** |
| Propulsion | 0.16±0.03 | **0.22±0.05ᵡ** | **0.26±0.08ᵡᶲ** | **0.12±0.02ᵡᶲ** | **0.18±0.07ᶲᵑ** |

Note: Data are mean ± SD

Abbreviations: Absorption (Abs), Generation (Gen), Moment (Mom)

**Bold:** Significant values

**ᵡ** Significantly different from the AB limb (p < 0.0125)

**ᶲ** Significantly different from the contralateral limb of the same prosthetic condition (p < 0.05)

**ᵑ** Significantly different from the same limb of the ESR condition (p < 0.05)
